# The Impact of Phospholipid-Based Liquid Crystals’ Microstructure on Stability and Release Profile of Ascorbyl Palmitate and Skin Performance

**DOI:** 10.3390/molecules29133173

**Published:** 2024-07-03

**Authors:** Alenka Zvonar Pobirk, Robert Roškar, Marija Bešter-Rogač, Mirjana Gašperlin, Mirjam Gosenca Matjaž

**Affiliations:** 1 Faculty of Pharmacy, University of Ljubljana, Aškerčeva cesta 7, 1000 Ljubljana, Slovenia; alenka.zvonar-pobirk@ffa.uni-lj.si (A.Z.P.); robert.roskar@ffa.uni-lj.si (R.R.); mirjana.gasperlin@ffa.uni-lj.si (M.G.); 2 Faculty of Chemistry and Chemical Technology, University of Ljubljana, Večna pot 113, 1000 Ljubljana, Slovenia; marija.bester@fkkt.uni-lj.si

**Keywords:** lamellar liquid crystals, microstructure, ascorbyl palmitate, stability, skin delivery systems

## Abstract

The drug delivery potential of liquid crystals (LCs) for ascorbyl palmitate (AP) was assessed, with the emphasis on the AP stability and release profile linked to microstructural rearrangement taking place along the dilution line being investigated by a set of complementary techniques. With high AP degradation observed after 56 days, two stabilization approaches, i.e., the addition of vitamin C or increasing AP concentration, were proposed. As a rule, LC samples with the lowest water content resulted in better AP stability (up to 52% of nondegraded AP in LC1 after 28 days) and faster API release (~18% in 8 h) as compared to the most diluted sample (29% of nondegraded AP in LC8 after 28 days, and up to 12% of AP released in 8 h). In addition, LCs exhibited a skin barrier-strengthening effect with up to 1.2-fold lower transepidermal water loss (TEWL) and 1.9-fold higher skin hydration observed in vitro on the porcine skin model. Although the latter cannot be linked to LCs’ composition or specific microstructure, the obtained insight into LCs’ microstructure contributed greatly to our understanding of AP positioning inside the system and its release profile, also influencing the overall LCs’ performance after dermal application.

## 1. Introduction

Long-term exposure of mammalian skin to ultraviolet radiation (UVR) induces oxidative stress by generating reactive oxygen species (ROS), acknowledged as the single major factor responsible for (premature) skin photoaging and various skin disorders and diseases, including skin cancer [1,2]. Excessive sun exposure compromises the integrity of various skin layers and induces a proinflammatory state in addition to DNA damage caused either by direct absorption of UVB by DNA skin cells or indirectly by the generation of ROS induced by UVA [3,4]. For preventing photoaging and skin cancer, it is, therefore, most important to reduce sun exposure and support the endogenous network of antioxidants offering skin protection against free radical damage [5,6]. Several studies have supported the benefit of the daily use of sunscreens to prevent solar skin damage, as reviewed by A. Jesus et al. [7]. In addition, dermal application of antioxidants to neutralize free radicals is an effective shielding strategy against the adverse effects of UVR [8,9].

Ascorbyl palmitate (AP) is an amphiphilic derivative of ascorbic acid widely used as an antioxidant-active substance in pharmaceutical and cosmetic formulations to combat skin photoaging, as reviewed previously [10]. Antioxidants formulated in classical skin care products have some common problems in their efficacy relating to their physicochemical and biopharmaceutical properties, e.g., low solubility, poor permeability, and instability [11]. Therefore, the implementation of novel carrier systems for efficient delivery and protection of antioxidants that can maximize their potential role in prophylaxis and therapy has been extensively investigated [12,13,14,15]. While novel drug delivery systems were initially developed to enhance the stability and solubility of incorporated active ingredients, nowadays, a key focus is on controlled drug release, enabling maintenance of therapeutic drug concentrations for a prolonged period of time and increased penetration of actives into the skin to reach cutaneous cells [16]. Various dermal delivery systems have previously been studied for providing stability and effectiveness of AP and other antioxidants, i.e., microemulsion and nanoemulsion, solid lipid nanoparticles and nanostructured lipid carriers, and liposomes [17,18,19,20,21]. The stability of AP was reported to depend on loading concentration, its location in the microemulsion in addition to the oxygen dissolved in the system, and light exposure [22]. Liquid crystals (LCs) with a lamellar structure seem to be among the most promising systems for dermal delivery of antioxidants to reduce the burden of UVR-induced skin disorders. They offer ideal consistency and thermodynamic stability in addition to great solubilization and sufficient stabilization potential while enabling modulation of their release and absorption characteristics [23,24]. The pronounced similarity of lamellar LCs with the intercellular lipid matrix of the stratum corneum and their skin-hydrating properties originating from interlamellar water that is less prone to evaporation when applied to the skin present additional benefits compared to other carriers [25,26]. The advantages presented that are linked to the wide drug delivery potential of lamellar LCs are fundamentally related to their microstructure. The latter is a result of the specific arrangement of hydrates and solvates of surfactants in the presence of water and/or oil phases, i.e., altering polar and nonpolar layers in the case of lamellar LCs [15,27,28]. In addition to lamellar, hexagonal and cubic LC structures can also be formed upon hydration or solvation of surfactants [27,29,30,31]. In keeping with this, lyotropic LCs may show diverse behavior regarding the stability and release of the solubilized drug; therefore, an insight into the structural characteristics of lyotropic LCs (with and without the incorporated drug) is of crucial importance to assess their drug delivery potential [32,33,34].

Clinical relevance for the treatment of aged skin was previously identified for lamellar LCs composed of lecithin and Tween 80 [35]. With diverse research fields involving a combination of the two amphiphiles, their intermediate structures and structural pathways remain a subject of research interest [36]. In addition, cubic and two lamellar mesophases were identified in the AP/water binary system depending on concentration and temperature [37], which increases the chance for microstructural changes of lamellar LCs upon incorporation of amphiphilic AP. Moreover, LCs are exposed to (moderate) temperature changes and some dilution upon application, which are both possible drives for phase conversions taking place. According to the literature, pH, light, magnetic field, additives, and the type of amphiphilic are also recognized as stimuli leading to the microstructural rearrangement of LCs [38,39,40]. Phase transitions can influence the drug diffusion and release profile as well as the overall skin performance of LCs, so the knowledge of microstructural rearrangements driven by temperature, water content, and drug loading is important for the development of dermally applicable drug delivery systems from this point of view [40]. In contrast to the simplicity of their preparation, the characterization of the lyotropic LC microstructure is far from trivial and requires a combination of several techniques.

The aim of the present study is to evaluate the lamellar LCs as a dermal delivery system for AP. Physiologically compliant lamellar LCs for dermal delivery of AP composed of isopropyl myristate (IPM)/Tween 80/lecithin/water were previously developed by our group [13] and studied for phase behavior and structural features as a function of temperature and water content in lyotropic LCs, positioned on the same dilution line as the pseudoternary phase diagram [41]. As an extension of previous work, the drug delivery potential of developed lamellar LCs for AP was assessed in the present study. Systems located along the dilution line were thus investigated regarding their ability to stabilize AP and drug release characteristics in addition to the pig’s ear skin performance. As the amphiphilic moiety AP was expected to contribute to microstructural transitions of LCs along the dilution line, structural alterations possibly taking place due to its incorporation were investigated by polarization microscopy, small-angle X-ray scattering (SAXS), differential dynamic calorimetry (DSC), and rheology analysis. Obtaining insight into structural transitions is expected to contribute greatly to our understanding of drug positioning inside the system and its release profile, influencing overall skin performance.

## 2. Results and Discussion

While dermal formulations intended for active skin care and/or therapy usually comprise active ingredients incorporated in conventional, either semi-solid or liquid formulations, an innovative approach is to formulate an advanced delivery system in order to utilize its unique advantages that would support drug action and together facilitate patient-friendly treatment and improved therapeutic outcome(s). As lyotropic LCs and, in particular, lamellar lyotropic LCs are considered the most suitable system, their drug delivery potential for AP (i.e., stability, release profile, skin performance) was explored in relation to their microstructure. The formation of lamellar phases is governed by suitable self-assembly of hydrated or solvated amphiphiles; therefore, all constituents must be carefully selected. In our case, the formation of lipid bilayers is favored by lecithin’s critical packing parameter, i.e., 0.5 to 1, while assembly into spherical micelles is characteristic of Tween 80 due to its considerably lower critical packing parameter being 0.07. While the microstructure of unloaded LCs positioned on the same dilution line was studied in our previous research [41], the incorporation of AP is also expected to influence their arrangements due to its amphiphilic character. To assess phase transitions possibly relevant for the drug delivery potential of AP, a combination of complementary characterization techniques was used.

### 2.1. Ascorbyl Palmitate Stability

Although AP is widely used in topical formulations as a more stable oil-soluble derivative of vitamin C, it was reported that the molecule is still susceptible to hydrolysis taking place in finished products as solutions and emulsions, even when employed in suitable gel-like emulsions with high viscoelastic properties that may improve its chemical stability [42]. Lamellar LCs have previously been suggested as suitable carriers for dermal delivery of AP, also from a stability point of view [23,43].

To accurately assess the stability of AP in tested formulations, we first optimized and validated the high-performance liquid chromatography (HPLC) method, initially developed in our previous study [43]. Prior to injection, tested AP-loaded LCs were diluted with methanol to obtain an AP concentration of approximately 40 mg/L. The data on the repeatability of the AUC for AP obtained upon two subsequent injections of the same sample revealed the need for stabilizing the AP in prepared methanol solutions. Its stability was improved by the addition of ascorbic acid at a concentration of 200 mg/L. With 96.2% of nondegraded AP upon 24 h of storage (compared to 65.9% in reference methanol solution), ascorbic acid was confirmed as the most efficient for stabilizing AP in HPLC samples among tested antioxidants and solvents (i.e., ascorbic acid, EDTA, BHT, methanol, and acetonitrile) (data are presented in Supplementary Materials). The HPLC method was then validated to confirm that it is accurate, reproducible, and sensitive within the specified analysis range. The specificity of the developed procedure was confirmed, as no other component of the tested samples or solvents had the same retention time as AP (i.e., approximately 4.8 min). The RSD of the AUC for tested samples prepared in five parallels was below 1%, thereby confirming the repeatability of the method, whereas its accuracy tested in three parallels was confirmed to be 100.3%, with a low RSD. The standard curve was linear with a correlation coefficient (r^2^) of 0.9996, over the range of 5–500 mg/L for AP. The HPLC method validation data are presented in Supplementary Materials.

**Figure 1:**
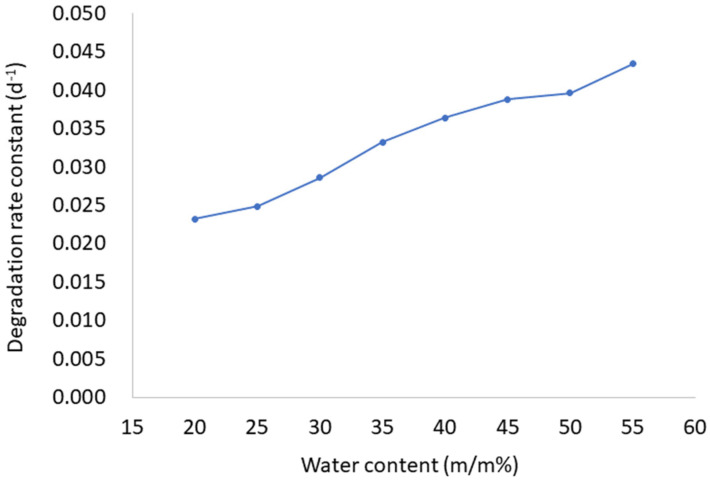
The first-order degradation rate constant of AP as a function of water content (m/m%) in LCs.

The stability of AP in prepared LC formulations was tested during 8 weeks of storage under controlled conditions. As evident from Table 1, AP stability is affected by water content in LCs. AP is more stable in samples with the lowest water ratio (LC1-AP–LC5-AP) than in more diluted samples (LC6-AP–LC8-AP). The AP stability decreased with increasing water content over the 28 days of the study. After 4 weeks, the amount of nondegraded AP in the sample LC1-AP (with the lowest water content) was 52%, while in the most diluted LC8-AP with the highest water content, only 29% of AP was detected. A possible explanation is a higher amount of interlamellar water, allowing the dissolution of more oxygen, which leads to the degradation of AP positioned at the interface between the polar hydrophilic heads of lecithin and Tween 80 and the interlamellar space. The stability of AP is further affected by the viscosity of the tested formulations. Although LC1-AP and LC2-AP contain a higher amount of water as self-microemulsifying drug delivery systems (SMEDDSs, i.e., the anhydrous system used for comparison), AP was more stable in LCs due to their higher viscosity (approximately 47 (LC1-AP) and 19 (LC2-AP) Pa*s compared to 8 Pa*s (SMEDDS) at 25 °C, respectively), limiting the diffusion of oxygen and thus AP oxidative exposure and degradation [43]. Likewise, AP was less stable in water-in-oil microemulsions (W/O MEs), being less viscous as compared to LCs. The trend was similar after 56 days of storage. The AP degradation during the first 28 days followed the first-order kinetic (the Pearson’s coefficients value was above 0.98 with the exception of LC1-AP (0.959) and LC2-AP (0.974), and the degradation rate constant increased with higher water content (Figure 1).

Although the stability of AP in LCs is higher, as reported by Špiclin et al. [22], who determined 19% of remaining AP in oil-in-water microemulsions and ~13% in water-in-oil microemulsions, both loaded with 1% AP and stored at 22 ± 1 °C for 28 days, it is inferior to the study of Üner et al. [21]. They reported that 48% (nanostructured lipid carriers), 59% (solid lipid nanoparticles), and 50% (nanoemulsion) of nondegraded AP were detected in samples loaded with 1% AP after 3 months of storage at 40 °C. As for our study, below 10% of AP was nondegraded after 56 days, yet again with the exception of LC1-AP and LC2-AP, with 14.8% and 10.6% of AP nondegraded, respectively. Therefore, aiming to improve the AP stability in LCs, they were co-loaded with 1% AP and 1% vitamin C. The results of the preliminary study indicate that for LC1-AP, the percentage of nondegraded AP increased from 13 to 36% after 56 days of storage as a result of the stabilizing effect of vitamin C. As LC1 with a lower water content shows a high solubilization capacity for AP, it could be loaded in higher concentrations, which was identified as beneficial as well (after 56 days of storage of LC1-AP loaded with 5% AP, the amount of nondegraded AP was ~33%). Both approaches tested were proven promising to further address AP (in)stability in LCs’ as well as in other (phospho)lipid-based formulations.

### 2.2. In Vitro Release Profile of Ascorbyl Palmitate

**Figure 2:**
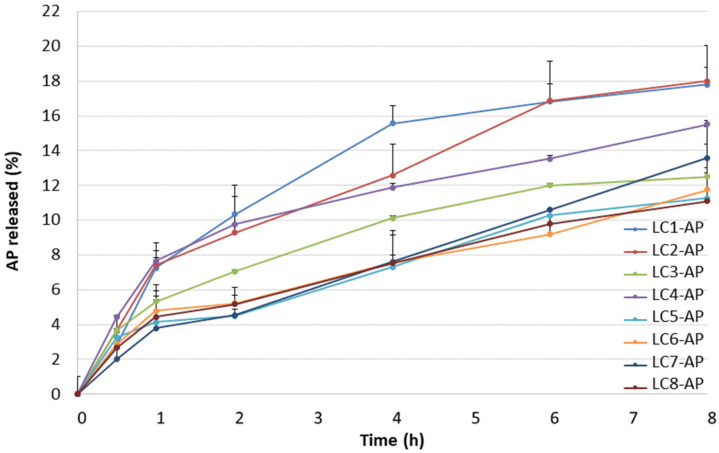
The release profiles of AP from LCs.

Due to specific microstructure and specific rheological characteristics, lamellar LCs are recognized as better alternatives to conventional emulsion systems, not only in terms of stability but also in terms of controlled release and moisturizing ability. Alternation of LC microstructure can occur due to dilution with physiological fluids, i.e., with water on the skin surface in cases of dermal application, which consequently implies different release rates. Information on the diffusion of an active ingredient from the vehicle can be provided by in vitro release studies through the artificial hydrophobic membrane, which depends on the physical–chemical properties of components, the internal structure of the vehicle, and the interaction between the drug and the vehicle [44,45,46]. To assess the drug release profile, testing conditions were optimized with regard to the pore size of the acetate cellulose membrane (i.e., 0.2 μm vs. 0.45 μm) and composition of the release medium (i.e., methanol/ultrapure water ratio of 85/15, 70/30, and 50/50 with ascorbic acid added as a stabilizer in 200 mg/L concentration). The release profiles of AP from LCs 1–8 through the artificial membrane with higher pore size into the medium with the highest methanol content are shown in Figure 2. AP release was characterized by two parameters: the amount released after 8 h and the rate of drug release.

The highest amount of AP was released from LC1-AP and LC2-AP (~18% after 8 h). As they differ in microstructure, as confirmed by structural characterization, this indicates the importance of water content between layers. Namely, water content determines the state of interlamellar water as a result of diverse interactions with amphiphilic molecules, i.e., lecithin and AP, in the case of AP-loaded LC. The similarity of both profiles was also proposed by the values of dissolution profile difference factor f_1_ (i.e., 8) and similarity factor f_2_ (i.e., 89). For two dissolution profiles to be considered similar, f_1_ should be between 0 and 15, whereas f_2_ should be between 50 and 100 [47].

The intermediate amount of AP was released from samples LC4-AP, LC7-AP, and LC3-AP. For samples LC3-AP and LC4-AP, the most distinct Maltese crosses typical of lamellar mesophases were observed by polarized light microscopy in addition to LC5-AP. So, parallel movements of layers that ease the AP diffusion most likely ease the AP release from samples LC3-AP and LC4-AP.

The lowest amount of AP was released from most diluted samples, LC5-AP, LC6-AP, and LC8-AP, with the exception of LC7-AP. This effect can be attributable to the pronounced swelling of lecithin, resulting in increased viscosity and increased interlayer spacing due to higher water content (also indicated by a reduction in Maltese crosses), altogether hindering the AP diffusing from the system. AP release profiles for LC5-AP and LC6-AP are also most similar to LC8-AP, having the lowest values of difference factor f_1_ (6 and 5, respectively) and highest values of similarity factor f_2_ (98 for both pairs) among all profiles.

The AP release kinetics were analyzed using zero- and first-order kinetics as well as the Korsmeyer–Peppas, Higuchi, and Hixon–Crowell models (Table 2). The calculated Pearson’s coefficients (in the range of 0.9640–0.9939) indicate the best fit for the Higuchi model, suggesting AP release by diffusion from all samples, with the exception of LC3-AP and LC7-AP. This agrees with Martiel et al. reporting caffeine release profiles from cubic and lamellar LCs fitting to the Higuchi model [34]. LC3-AP and, in particular, LC7-AP show a slightly better fit with the Korsmeyer–Peppas model and first-order kinetic, respectively. In the case of LC3-AP, the value of diffusion exponent *n* was greater than 0.89, suggesting the supercase II transport release mechanism, whereas a concentration-dependent release mechanism was proposed for LC7-AP. This is in line with the AP release profiles for LC3-AP and LC7-AP that stood out from other samples positioned on the same dilution line.

### 2.3. In Vitro Skin Performance

The excellent skin performance of the lamellar LCs is largely related to the similarity of their microstructure to the intercellular lipid matrix of the stratum corneum [48]. The biological acceptability of the lamellar LCs was confirmed on isolated keratinocytes [35], while in the present study, the performance of LCs was evaluated by measuring their influence on barrier function and hydration level of pig’s ear skin in vitro.

Application of all LCs resulted in lower transepidermal water loss (TEWL) values measured 30 min and 90 min after they were removed from the skin (Table 3). More precisely, after 30 min between 1.2-fold (LC1) and 1.04-fold (LC7), lower TEWL values were observed as compared to the basal measurements, though not statistically significant. A similar trend was also observed after 90 min, where LC1 and LC5 performed best. This allows us to confirm the barrier-strengthening effect of LCs; nevertheless, it cannot be linked to their composition (e.g., water content) or specific microstructure, as the differences among systems were not significant. In addition to intact barrier function, proper hydration of the epidermis is important to support epidermal homeostasis and maintain skin health [49]. In agreement with decreased TEWL, improved skin hydration was determined for all LCs at both measurement time points. After 30 min, the best skin hydration values compared to basal measurements were observed for LC1 (1.9-fold increase) and LC2 (1.6-fold increase (*p* < 0.05), whereas the lowest effect was observed for LCs with higher water content (LC5-LC8). After 90 min, the skin-hydrating effect of LCs is less pronounced; nevertheless, the observed trend is still visible. The prolonged moisturizing ability of LCs, especially for LC1–LC4, among which a significant, 1.7-fold (*p* < 0.05) improvement was observed for LC3, can be explained by their internal structure, with bulk water present in the interlamellar space together with loosely or intermediately bound water of the second hydration layer, as confirmed by DSC analysis, which results in prolonged release. This is in line with the lower water loss rate of LC emulsion observed by Bing et al., who reported improved moisturizing properties in addition to the slow release and promoted penetration effect of LC emulsion as compared to conventional emulsion [50]. In addition, lipophilic components of LCs present an emollient effect and decrease TEWL, thereby supporting water retention within the stratum corneum. In this regard, further clinical assessment for a final comprehensive appraisal of tested LCs would be applicable, especially as recently beneficial short- and long-term effects of hempseed or flaxseed oil-based lamellar LCs on the skin barrier function of healthy adult subjects were reported [26].

### 2.4. Structural Characterisation of LCs

The development and detailed structural evaluation of lecithin-based lamellar liquid crystals positioned on the same dilution line were reported in our previous research [41]. This study involves the evaluation of stability, release profile, and skin performance to conceptually upgrade our earlier work aiming to develop an advanced dermal formulation to combat skin photoaging. In this regard, structural characterization of AP-loaded LCs was performed in order to support and correlate the obtained results with the microstructure of the samples investigated. As the microstructure of lyotropic LCs is temperature-dependent, structural characterization was performed at specific targeted temperatures to support the stability and skin performance studies. More precisely, samples were tested at 32 °C and 37 °C, mimicking dermal delivery in addition to ambient conditions representing the storage temperature.

#### 2.4.1. Polarized Light Microscopy Investigations

**Figure 3:**
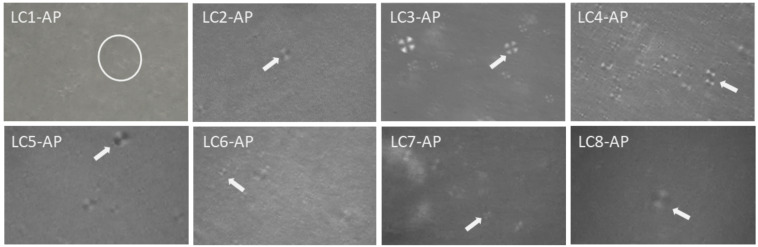
Polarized light microscopy photomicrographs of representative LC samples; encircled are fan-shaped textures (LC1-AP), while white arrows point to Maltese crosses observed for LC2-AP to LC8-AP.

With AP incorporated in lyotropic LCs as an active ingredient with amphiphilic character, especially when considering its self-assembly property [37], an alternation of microstructure could take place. Visualization and preliminary microstructure identification at 25 °C of AP-loaded LC1-LC8 were performed using cross-polarized light microscopy. Clearly seen Maltese crosses confirmed the lamellar microstructure for AP-loaded LC samples (Figure 3) apart from LC1-AP. Even though not pronounced, fan-shaped structures imply hexagonal mesophases for LC1-AP that are most likely locally distributed. Considering that the following dermal application formulation is being subjected to physiological dilution with water passing through the skin as TEWL, it could be postulated that the LC1-AP system would likewise possess lamellar structure as observed for all other AP-loaded LCs.

#### 2.4.2. SAXS Analysis

**Figure 4:**
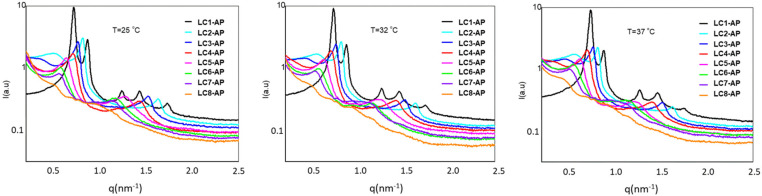
Scattering curves for the LC1-AP and LC8-AP samples at 25 °C, 32 °C, and 37 °C.

The SAXS measurements were performed for AP-loaded LCs at three predetermined temperatures, with corresponding SAXS spectra shown in Figure 4. Ordered lamellar microstructure is reflected in scattering vector q in the ratio q_1_:q_2_ = 1:2 observed for all AP-loaded LCs apart from the samples with either the lowest or the highest water content, i.e., LC1-AP or LC8-AP, respectively. As for the samples that exhibited lamellar structure, a strong intensity of SAXS scattering peaks was observed. As their intensity or width remained practically unchanged at temperatures relevant for in vitro release testing, it is reasonable to conclude that the AP release takes place in the ordered bilayer structure. A small and wider peak with low intensity was randomly detected before the first scattering peak for LC2-AP and LC3-AP (and only at 37 °C for LC4-AP), most likely arising from the co-existence of micellar aggregates. The different microstructure of LC1-AP being anticipated based on prelaminar visualization was indeed confirmed by five distinctive scattering peaks with ratios inconsistent for either hexagonal or cubic arrangements and remaining consistent for all temperatures tested. On the other hand, no distinct peaks were observed for LC8-AP, indicating a lack of lamellar arrangement; nevertheless, observed Maltese crosses by polarized light microscopy could indicate local areas of formed lamellae. Additionally, the interlayer spacing *d* was calculated according to the results presented in Table 4. If the spacing for LC1-AP structures remained practically unaltered at temperatures tested, a continuously increasing interlayer spacing ranging from 7.74 nm for LC-AP2 to 11.24 nm for LC7-AP at 25 °C was observed along the dilution line, coinciding with the “lamellar” swelling law [38,51]. The same trend was followed by increasing temperatures, yet repeated distances were slightly increased. Lack of LC structural organization in the case of LC8-AP (therefore, no repeated distance could be determined) implies that systems undergo phase transition due to dilution, with 50% (m/m) water being the maximum amount that still holds lamellar arrangement. This is in line with DSC results with higher amounts of bulk water detected for more diluted systems and, in particular, with elevated AP release from LC7-AP with presumably more loose lamellas due to excess free water between the polar heads of lecithin and AP molecules. 

#### 2.4.3. DSC Analysis

As reported previously, different types of water have been detected in surfactant-based microstructures like lyotropic LCs, as their behavior is sensitive to the presence of adjacent interfaces of varying types [52,53]. Different physicochemical characteristics of strongly bound (nonfreezable) water of the first hydration layer strongly interacting with the polar heads of surfactants, loosely or intermediately bound (freezable) water of the second hydration layer, and the free water present in the bulk layer influence not only the thermal behavior of LCs (e.g., intermediately bound water crystallizes at a lower temperature as bulk water and evaporates more slowly) but also their skin performance (e.g., the duration of moisturization effect) [50]. The amount of bulk water in LCs is expected to increase along the dilution line by increasing the distance between the lamellae. The incorporation of drugs with amphiphilic characters like AP may show the opposite effect, though. The state of water in prepared systems was likely linked to phase changes in lyotropic LCs, and their performance was thus determined by DSC analysis.

**Figure 5:**
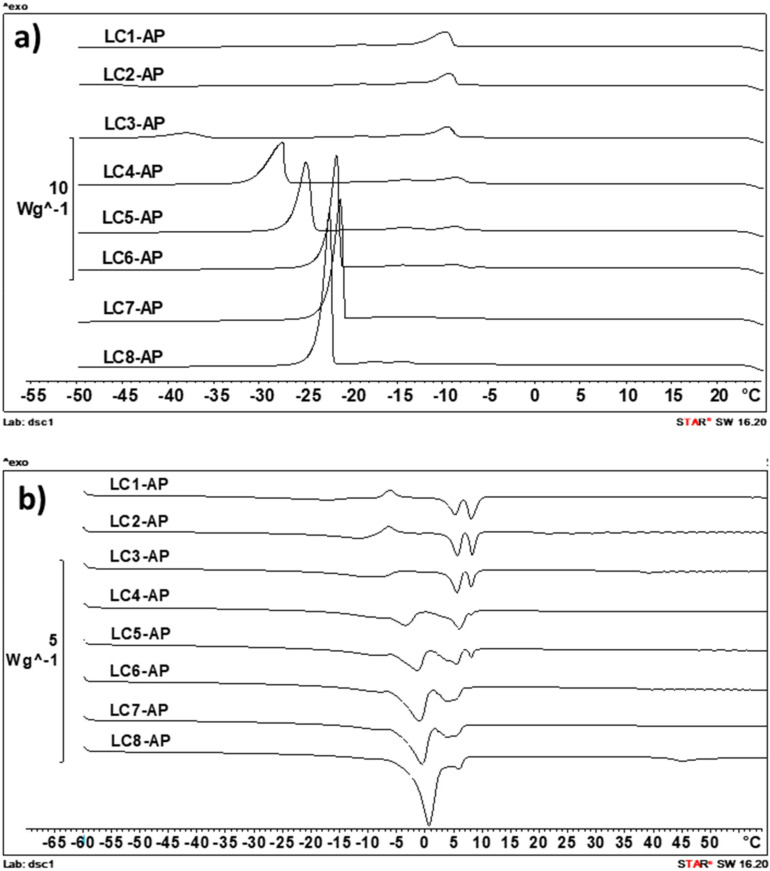
Differential scanning calorimetry (DSC) cooling curves (5 K/min) (**a**) and heating curves (5 K/min) (**b**) for the AP-loaded LCs.

The DSC scans of samples were performed with cooling/heating rates of 2, 5, and 10 K/min, and the DSC thermograms obtained by the intermediate temperature increasing/decreasing rate are presented in Figure 5. This scanning rate was identified as optimal with regard to sensitivity and selectivity and also enabled the best comparison with data obtained for unloaded LC counterparts presented in our previous study [41].

In the cooling curves of all LC samples (Figure 5a), the solidification of IPM is clearly visible, as indicated by the “triple exothermic peak” between −7 °C and −17 °C, most probably indicating the solidification of different polymorphs [53]. The other exothermic event visible in cooling curves of samples with water content above 25–30% (m/m) is its freezing around −24 °C (in LC7-AP and LC8-AP with 50 and 55% (m/m) water) and −43 °C (in LC3-AP with 30% (m/m) water). In LC2-AP with 25% (m/m) water, its crystallization was only visible at a cooling rate of 2 K/min (−47 °C), while in LC1-AP, it could not be detected. The cooling curves of AP-loaded samples thus confirmed the presence of nonfreezable interlamellar water (the first hydration layer) and two types of freezable water, presenting the second hydration layer that keeps the degree of freedom necessary to form ice-like hydrogen bonds and bulk water. With increasing amounts of water, the crystallization peak of immediate bund water increases in area and shifts towards higher temperatures (visible in samples LC2-AP to LC5-AP) until the freezing peak of free water can be seen in samples LC6-AP to LC8-AP. Presumption on the co-existence of bound water with bulk water in samples LC6-AP to LC8-AP was to some extent confirmed by measurement of pure double distilled water, where a similar peak at approximately −21 °C was also observed (at cooling rate 5 K/min), indicating freezing of supercooled water. Regarding the state of water present in AP-loaded lyotropic LCs, the DSC heating curves are, for the most part, in agreement with conclusions made based on DSC cooling curves. In agreement with Kodama and Aoki [54], the ice obtained from freezable interlamellar water begins to melt at temperatures as low as around −40 °C (for samples with the lowest water content) or −30 °C (for samples containing above 30% (m/m) water) and continues to melt up to above 0 °C, whereas the ice derived from bulk water melts in a narrow temperature range around 0 °C (visible in LC5-AP to LC8-AP).

It was expected for AP to distribute into bilayers due to its amphiphilic nature, resulting in a decreased amount of freezable water in LCs in the presence of AP as compared to their unloaded counterparts [42]. Namely, AP molecules are expected to present additional polar headgroups interacting with water molecules in an intrabilayer of lamellar LCs or rod-like aggregates (i.e., micelles) in the separation zone of the hexagonal phase (as seen for sample LC1-AP). Based on the DSC cooling curves, the proposed hypothesis could be partially confirmed for sample LC2-AP, for which the water freezing peak can only be detected by the lowest cooling rate of −2 K/min (not in all parallels, though), and samples LC3-AP and LC4-AP, for which the water freezing peak (presented as T_onset_) was shifted towards lower temperatures upon incorporating AP (from approximately −32 °C to −38 °C for LC3-AP and from approximately −29 °C to −37 °C for LC4-AP), while this was not the case in samples with water content above 40% (m/m).

#### 2.4.4. Rheological Behavior

While rotational measurements present an important quality control tool for all dermally applicable pharmaceutical systems by giving information on their flow properties and features under applied stress [55], more specific information on the network structure of the liquid crystal phases can be obtained from dynamic strain sweep measurements and oscillatory shear frequency sweep measurements. All samples tested had relatively high consistency and exhibited a strong decrease in viscosity with the growing shear rate at all temperatures tested, confirming shear-thinning behavior typical of pseudoplastic systems (viscosity flow curves for LC1-AP to LC8-AP are a part of the Supplementary File). In lamellar LCs, such behavior originates from their smectic structure, where parallel layers slide over each other with relative ease during shear [56,57,58].

At the lowest measured shear stress (2 s^−1^), the viscosity of LCs decreases with increasing temperature (Table 5). While the gradual increase in water content along the dilution line was linked with increased viscosity at 25 °C for unloaded samples (with the exception of the sample with the highest water content) [41], the viscosity dependence of LCs on water content is not so straightforward in the presence of AP. The sample with the lowest water content (LC1-AP) that was identified as hexagonal mesophase shows considerably higher viscosity than subsequent samples (from LC2-AP to LC4-AP at 25 °C or up to LC6-AP at higher temperatures) and is also the only AP-loaded sample that is more viscous than its unloaded counterpart [41]. Some microstructural phenomena can additionally be observed in LC4-AP (at all temperatures tested), LC6-AP (only at 25 °C), and LC8-AP (at all temperatures tested), all having a lower viscosity than the previous sample on the dilution line. Similar phenomena observed in the unloaded counterpart of LC8-AP were related to a phase transition into the micellar phase taking place due to an increase in water content as the main drive, associated with pronounced dilution leading to swelling of the structures due to the incorporation of water between lamellas [41]. The most pronounced impact of AP incorporation on the rheological behavior of LCs was observed for LC4-AP, which is considerably less viscous than LC3-AP. Opposite results were observed in unloaded counterparts, with LC4 being more than twice as viscous as LC3 [41]. In general, the viscosity of samples LC4-AP and LC6-AP was also most affected by the temperature increase. In agreement with dilute lamellar phases typically occurring in relatively narrow ranges of temperature, temperature increases the long-range undulating repulsion between layers, and thus, the free energy of the system can be increased by the mechanism of decreased bending modulus [39]. In agreement with the well-known fact that the lamellar liquid crystalline phase shows lower values of the rheological functions than other lyotropic phases detected in nonionic surfactant/water phase diagrams [59], systems LC2-AP to LC6-AP may be classified as lamellar LCs.

**Figure 6:**
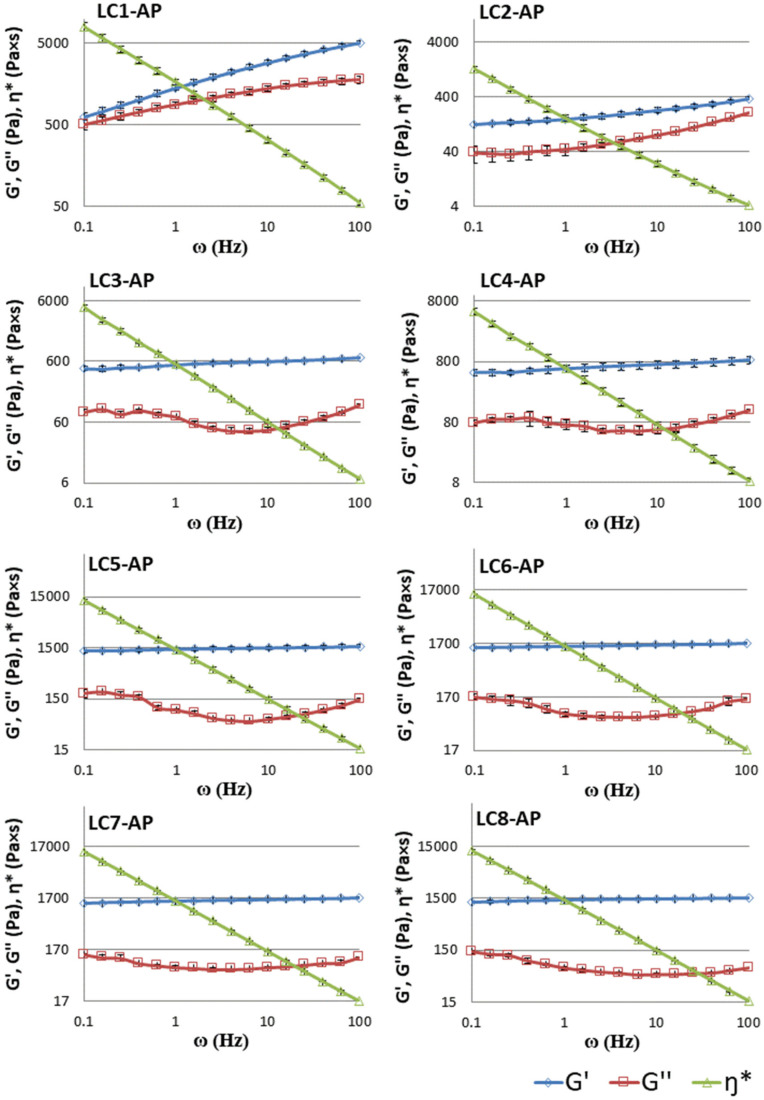
Storage modulus (G′, -◊-), loss modulus (G″, -□-), and complex viscosity (η*, -∆-) for the LC1-AP to LC8-AP samples as a function of frequency (ω) at a stress of 10% at 25 °C. Data are presented as means ± S.D. (*n* = 3).

The linear viscoelastic properties of LCs were determined by means of frequency sweeps inside the linear viscoelastic region to obtain more information on the network structure of LCs. As observed in Figure 6, almost constant values of the storage modulus *G*′, showing only a slight increase with increased frequency, and a clear minimum in the loss modulus G″ can be detected for systems LC3-AP to LC7-AP (partially also in LC2-AP and LC8-AP with only a slightly pronounced minimum in G″). At the same time, the complex viscosity η* drops linearly as a function of frequency for all systems tested. The presented rheological behavior is typical of lamellar phases and other gel-like structure systems that are also characterized by higher values of storage modulus in a wide range of frequencies [60,61,62]. Contrary to other LCs tested, different rheological behavior was observed for sample LC-AP1, which had the lowest water content among all samples. Both dynamic moduli (*G*′ and *G*″) enhanced with increasing frequency, with storage modulus *G*′ having a greater slope than loss modulus *G*″ (for LC1-AP). The observed rheological pattern is representative of the hexagonal LC phases [63], usually showing traits of the general Maxwell model, in which the values of the dynamic moduli increase with increasing frequency with different slopes [60]. The obtained data correspond well with the SAXS analysis of LC-AP1.

**Figure 7:**
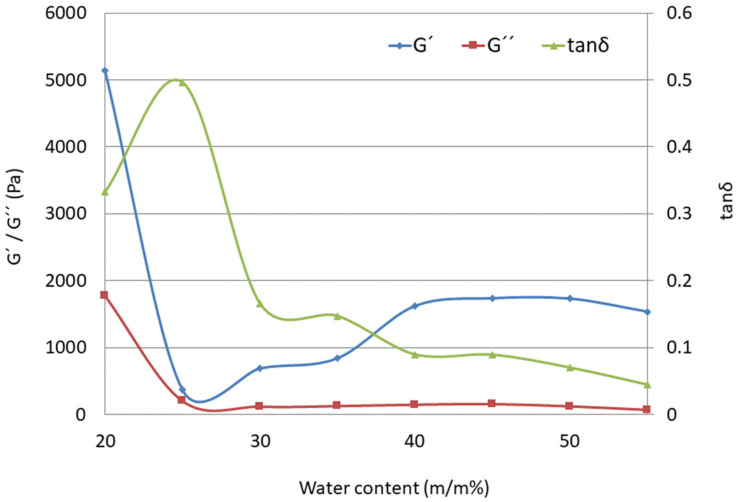
Storage modulus (G′, -◊-), loss modulus (G″, -□-), and loss tangent (tanδ, -∆-) as functions of water content (m/m%) for the LC1-AP to LC8-AP samples (at 100 Hz frequency).

According to the results presented in Figure 7, the loss modulus *G*″ is practically independent of water content, while the storage modulus *G*′ increases with a higher water ratio. The ratio between *G*′ and *G*″ (tan δ) below 1 is suggestive of an elastic gel structure [63]. As presented in Table 5, the elasticity of LC systems increases with increasing water-to-surfactant ratios (from LC2-AP to LC8-AP), as seen from the decrease in tan δ at a frequency of 100 Hz. Based on the values of the tan δ samples, LC3-AP to LC4-AP and LC5-AP to LC7-AP show the most comparable characteristics. As expected, tan *δ* was found to be significantly larger for LC1-AP (0.333) and LC2-AP (0.497) than that for the lamellar phase (between 0.166 for LC3-AP and 0.070 for LC7-AP or 0.044 for LC8-AP), which implies a more viscous gel structure and a completely different rheological pattern, i.e., altogether, a different microstructure. In agreement with the aforementioned data and DSC results, the tan δ values of LCs were not influenced in a straightforward way upon incorporation of AP. While systems with intermediate water content (LC5-AP and LC6-AP) seem least affected, the most pronounced changes were observed in systems with the lowest (LC1-AP and LC2-AP) and highest water content (LC8-AP).

## 3. Materials and Methods

### 3.1. Materials

Isopropyl myristate (IPM) of declared purity equal to or above 90%, and ascorbyl palmitate (AP) were obtained from Sigma-Aldrich, St. Louis, MO, USA, while Tween 80^®^ (polyoxyethylene (20) sorbitan monooleate) and 1-Butanol were purchased from Merck KGaA, Darmstadt, Germany. Soybean lecithin (Lipoid S-100^®^; not less than 94% m/m phosphatidylcholine content) was provided by Lipoid GmbH, Ludwigshafen, Germany.Bidistilled water was used throughout the experiments.

### 3.2. Methods

#### 3.2.1. Sample Preparation

Representative samples of AP-loaded LCs, whose composition is presented in Table 6, were further characterized. Samples were prepared by mixing appropriate amounts of IPM, Tween 80, and lecithin to form a homogeneous mixture in which 1% (m/m) of AP was dissolved. Water was added afterwards during continuous stirring to form lyotropic LCs.

For the AP stability study, two additional samples were tested, i.e., SMEDDS and W/O ME. SMEDDS was obtained by blending the IPM and surfactant mixture (Tween 80/lecithin) at mass ratio 7/3, while in case of W/O ME IPM (25.25%) and surfactant mixture with butanol as cosurfactant (Tween80/lecithin/butanol at mass ratio 1/1/2; 58.90%) were homogenously mixed and then diluted with bidistilled water (14.85%). For both samples, AP (1%) was dissolved in an oil–surfactant mixture.

For skin performance testing, the LC samples were prepared without AP incorporated (LC1-LC8) following the same procedure and keeping the mass ratio between the components (Tween 80/lecithin/IPM/water), as reported in Table 6.

#### 3.2.2. Stability Study

The tested samples were stored for 8 weeks at 40 °C, 75% relative humidity, and protected from light in glass containers. At predetermined time points (0, 1, 7, 14, 28, 47, and 56 days), 100 mg of each sample was diluted to 25 mL with methanol containing ascorbic acid at a 200 mg/L concentration as a stabilizer. The concentration of AP in the tested samples was determined by HPLC analysis. Measurements were performed by the Agilent 1200 series HPLC system. The stationary phase was a 125 × 4 mm column packed with 5 μm Nucleosil C18, and the mobile phase was a mixture of methanol–acetonitrile–0.02 M phosphate buffer of pH 3.5 (75/10/15). The volume of injection was 20 μL, the flow rate was 1.5 mL/min, and the wavelength of UV detection was 254 nm. All analyses were performed at 25 ± 1 °C.

#### 3.2.3. In Vitro Drug Release Study

AP release through a hydrophilic cellulose acetate membrane was determined with a Franz diffusion cell (*n* = 4) with a diffusion area of 0.785 cm^2^ at 25 °C. A total of 9 mL of receptor medium (methanol and ultrapure water mixed at different ratios) was used, and 400 mg of tested AP-loaded LC1-LC8 was applied on the donor side. A total of 800 μL aliquots of the receptor medium were collected at predetermined time intervals (30 min, 1 h, 2 h, 4 h, 6 h, 7 h, and 8 h) and replaced by fresh medium. If the samples were turbid, they were diluted with receptor medium prior to the HPLC analysis that was applied to determine the AP content of the collected samples. Drug release was expressed by the amount of released AP (%) as a function of time.

#### 3.2.4. Skin Performance Testing

Skin performance testing was evaluated in vitro on pig’s ear skin mounted on Franz diffusion cells (*n* = 4). During the experiments, the temperature was kept at 22 ± 1 °C and the relative humidity between 40 and 60%. The receptor chamber was filled with physiological fluid (0.9% aqueous solution of NaCl), and the pig’s ear skin was placed between the donor and receptor compartments on the stratum corneum side. Prior to the application of LCs, the skin was allowed to temperate for 1 h; namely, the temperature of the receptor compartment was kept at 37 °C, establishing a temperature gradient with ambient room temperature, resulting in a skin temperature of 32 °C. Then, approximately 20 mg of LC sample was accurately weighed and transferred on the skin into the donor compartment for 60 min under non-occlusive conditions. Almost 60 min after the application of LCs, they were gently removed from the skin surface, which was whipped with a cotton stick. The skin performance was assessed at ambient room conditions for 30 min and 90 min after LCs were removed from the skin surface. TELW was measured by an open chamber device (Tewameter^®^ TM 300 from Courage + Khazaka, GmbH, Germany). Prior to measuring the probe, it was preheated to 32 °C by the Probe Heater^®^ PR 100 (Courage + Khazaka, GmbH, Germany). Individual measurements lasted for 60 s, with one reading collected per second. The average of 10 consecutive readings with the lowest SD represented the TEWL value used for further analysis. TEWL values are presented as absolute values (g/hm^2^).

Afterward, skin hydration was assessed by the Corneometer^®^ CM 825 (Courage + Khazaka, GmbH, Germany) at the same time points. The results are given in Corneometer^®^ CM 825 arbitrary units (a.u.) as a mean value of six subsequent measurements for each Franz diffusion cell.

For TEWL and skin hydration, statistical analysis was carried out using an independent sample Student’s *t*-test at the 0.05 level of probability.

#### 3.2.5. Structural Characterization

The microstructure of AP-loaded LCs was evaluated using a set of techniques typically used for structural characterization of LC systems and previously reported by our group [23,41].

##### Polarizing Light Microscopy

The structure of the AP-loaded LCs was examined with a microscope with polarization using a Physica MCR 301 rheometer (Anton Paar, Graz, Austria) at 25 °C. The magnification used was 20×.

##### Small-Angle X-ray Scattering

An evacuated Kratky compact camera system (Anton Paar, Graz, Austria) with a block collimating unit attached to a conventional X-ray generator (Bruker AXS, Karlsruhe, Germany) equipped with a sealed X-ray tube (Cu-anode target type) operating at 35 kV and 35 mA and producing Ni-filtered Cu Kα X-rays with a wavelength of 0.154 nm was used for SAXS measurements. The AP-loaded LCs were transferred to a standard quartz capillary placed in a thermally controlled sample holder centered in the X-ray beam, with measurements performed at 25, 32, and 37 °C. The scattering intensities were measured with a linear position-sensitive detector (PSD 50 m, M. Braun, Garching, Germany), detecting the scattering pattern within the entire scattering range simultaneously. For each AP-loaded LC, five SAXS curves with a sampling time of 15,000 s were recorded and subsequently averaged.

The interlayer spacing *d* was calculated according to the equation:

(1) $$d=2\pi /q_{1}$$

where *q*_1_ is the scattering vector magnitude of the first reflection.

##### Differential Scanning Calorimetry

The DSC thermograms of AP-loaded LCs were recorded in duplicates or triplicates using a differential scanning calorimeter (DSC1 STARe System, Mettler Toledo, Switzerland). Approximately 10 mg of the sample was weighed precisely into a small aluminum pan. The empty, sealed pan was used as a reference. Samples were cooled from 20 °C to −60 °C (cooling rates: 2, 5, and 10 K/min), kept at −60 °C for 15 min, and then heated back to 20 °C (heating rates: 2, 5, and 10 K/min) under a stream of nitrogen at 50 mL/min. The DSC thermograms of the individual components (Tween 80, lecithin, IPM, and water) plus their binary and ternary mixtures were presented in our previous study [41].

##### Rheological Measurements

Rheological evaluation of AP-loaded LCs was performed using a Physica MCR 301 rheometer (Anton Paar, Graz, Austria) and the cone-plate measuring system CP50-2 with a conical disc diameter of 49.961 mm and a cone angle of 2.001°.

Rotational tests were performed at 25.0 ± 0.1 °C, 32.0 ± 0.1 °C, and 37.0 ± 0.1 °C, and the shear rate was increased from 2 to 100 s^−1^. The viscosity was calculated according to the following equation:

(2) η=τ/γ˙

where *τ* is the shear stress and γ˙ is the shear rate.

Oscillatory tests were performed at a constant temperature of 25.0 ± 0.1 °C to define the storage and loss moduli, which are calculated according to Equations (3) and (4):

(3) G′=(τ/γ)×cos δ

(4) G″=(τ/γ)×sin⁡δ

where *τ* is the shear stress, *γ* is the deformation, and *δ* is the phase shift angle, together with complex viscosity calculated according to Equation (5):

(5) η*=τ/(γ×ω)

where ω is the angular frequency.

In order to determine the linear viscoelastic region, the stress sweep measurements were first performed at a constant frequency of 10.0 s^−1^. Afterward, the oscillatory shear measurements were carried out as a function of frequency (0.1–100 s^−1^) at a constant amplitude (10%) chosen within the linear region.

## 4. Conclusions

In the present study, phospholipid-based LCs were evaluated as dermal delivery systems for AP. It has been established that loading AP at 1% (m/m) results in its incorporation within preformed structures. While the systems with the lowest and highest water content, i.e., LC1-AP and LC8-AP, exhibited distinctive structural characteristics, lamellar microstructure was observed for LC2-AP to LC7-AP. As confirmed by SAXS, DSC, and rheology analysis, their bilayer features were responsive to increasing water content, which was finally reflected in diverse AP stability and release profiles. In general, samples with the lowest water content (LC1-AP and LC2-AP) exhibited the best AP stability (up to 52% and 10 to 15% of nondegraded AP upon 28 and 56 days of storage, respectively) and faster API release (~18% in 8 h) as compared to the most diluted sample, LC8-AP (29% and 4% of nondegraded AP upon 28 and 56 days of storage, respectively, and up to 12% of AP released in 8 h). Then again, overall high AP instability in LCs, being below 10% after 56 days (with the exception of LC1-AP and LC2-AP), was improved by testing two stabilization approaches, i.e., the addition of vitamin C and increasing AP concentration.

Even though no straightforward relationship between microstructure and LCs’ skin performance can be given, LCs’ skin barrier-strengthening and hydrating properties are apparent, with up to 1.2-fold lower TEWL and up to 1.9-fold higher skin hydration values as measured on a porcine skin model in vitro. The prolonged moisturizing ability of LCs can, to some extent, be linked to their internal structure, with bulk water present in the interlamellar space together with loosely or intermediately bound water of the second hydration layer, which results in prolonged release.

While the LC platform at this point also comprises novel systems, namely liquid crystalline nanoparticles with great potential for targeting various skin disorders, bulk lyotropic LCs with semi-solid consistency and, when developed, simple and fast production and thermodynamic stability, continue to be of high biomedical relevance as dermal delivery systems. The results obtained within the presented study provide solid ground for the utilization of lamellar LC-based formulations for skin delivery, tailored regarding microstructure in order to attain appropriate stability and release of incorporated actives as well as skin performance.

## Figures and Tables

**Table 1:** The stability of ascorbyl palmitate (AP; %) incorporated in liquid crystals (LCs), self-microemulsifying drug delivery systems (SMEDDSs), and water-in-oil microemulsion (W/O ME) is presented as the mean value ± standard deviation (SD).

	% of Nondegraded AP over Time (Days)						
Sample	t = 0	t = 1	t = 7	t = 14	t = 28	t = 47	t = 56
LC1-AP	100.0	97.8 ± 0.5	75.7 ± 0.1	66.6 ± 0.1	52.1 ± 0.8	28.0 ± 0.3	14.8 ± 0.4
LC2-AP	100.0	101.6 ± 0.1	78.7 ± 0.0	67.0 ± 0.1	51.0 ± 1.1	19.8 ± 0.3	10.6 ± 0.1
LC3-AP	100.0	100.5 ± 0.5	78.9 ± 0.1	65.0 ± 0.3	45.6 ± 1.2	12.4 ± 0.1	6.4 ± 0.2
LC4-AP	100.0	100.4 ± 0.6	78.1 ± 0.4	64.7 ± 0.1	39.8 ± 0.1	4.7 ± 0.1	3.0 ± 0.1
LC5-AP	100.0	96.8 ± 1.2	73.6 ± 0.2	59.9 ± 0.1	35.9 ± 0.3	4.5 ± 0.0	2.7 ± 0.1
LC6-AP	100.0	97.6 ± 0.7	69.0 ± 0.7	56.7 ± 0.8	33.6 ± 0.4	6.7 ± 0.0	4.2 ± 0.1
LC7-AP	100.0	97.1 ± 1.0	67.5 ± 1.7	57.3 ± 0.4	32.5 ± 0.3	5.8 ± 0.2	4.2 ± 0.0
LC8-AP	100.0	94.5 ± 0.1	62.3 ± 0.6	49.7 ± 1.3	29.3 ± 0.2	6.7 ± 0.1	4.3 ± 0.0
SMEDDS	100.0	98.1 ± 18.2	53.2 ± 9.3	43.6 ± 0.8	30.1 ± 5.8	13.7 ± 0.6	10.0 ± 0.6
W/O ME	100.0	95.5 ± 1.9	68.7 ± 0.1	61.2 ± 0.2	40.1 ± 0.3	18.2 ± 0.5	13.4 ± 0.3

**Table 2:** Pearson‘s coefficients (r^2^) values for the AP release from LC1-AP to LC8-AP fitted to zero-order and first-order kinetic, and Higuchi, Korsmeyer–Peppas, and Hixon–Crowell models.

	LC1-AP	LC2-AP	LC3-AP	LC4-AP	LC5-AP	LC6-AP	LC7-AP	LC8-AP
Zero-order	0.8489	0.9064	0.8674	0.8248	0.9339	0.9209	0.9818	0.9237
First-order	0.8635	0.9214	0.8794	0.8432	0.9411	0.9298	0.9854	0.9325
Higuchi	0.9640	0.9890	0.9884	0.9714	0.9777	0.9840	0.9673	0.9939
Korsmeyer–Peppas	0.9171	0.9651	0.9933	0.9569	0.9430	0.9689	0.9819	0.9830
Hixon–Crowell	0.8587	0.9165	0.8755	0.8372	0.9388	0.9269	0.9843	0.9296

**Table 3:** Absolute transepidermal water loss (TEWL; first row for each LC) values and absolute skin capacitance values (second row for each LC) with changes from baseline 30 min and 90 min after a 60 min treatment with LC1–LC8.

Sample	Baseline	Absolute Value after 30 min	Absolute Value after 90 min	Change ^#^ from Baseline after 30 min	Change ^#^ from Baseline after 90 min	*p*-Value *	*p*-Value **
LC1	37.90 ± 5.68	30.87 ± 5.81	30.19 ± 3.92	−7.03 (18.6%)	−7.71 (20.3%)	0.12	0.06
	7.9 ± 5.0	22.6 ± 9.6	15.0 ± 3.6	+14.7 (186.1%)	+7.1 (89.9%)	0.06	0.09
LC2	33.36 ± 5.06	30.45 ± 3.41	28.14 ± 4.33	−2.91 (8.7%)	−5.22 (15.6%)	0.44	0.22
	6.1 ± 1.6	15.6 ± 4.2	11.0 ± 4.8	+9.5 (155.7%)	+4.9 (80.3%)	0.04	0.25
LC3	31.91 ± 5.97	27.73 ± 6.04	26.90 ± 7.67	−4.18 (13.1%)	−5.01 (15.7%)	0.35	0.33
	5.5 ± 4.2	11.8 ± 6.9	14.6 ± 5.9	+6.3 (114.6%)	+9.1 (165.5%)	0.16	0.03
LC4	28.29 ± 7.85	24.68 ± 7.06	23.75 ± 8.74	−3.61 (12.8%)	−4.54 (16.1%)	0.58	0.53
	15.4 ± 4.2	33.3 ± 7.9	29.6 ± 11.0	+17.9 (116.2%)	+14.2 (92.2%)	0.01	0.08
LC5	36.76 ± 4.10	32.49 ± 4.47	28.54 ± 5.44	−4.27 (11.6%)	−8.22 (22.4%)	0.27	0.08
	5.3 ± 1.6	6.0 ± 3.6	5.9 ± 3.7	+0.7 (13.2%)	+0.6 (11.3%)	0.75	0.79
LC6	31.51 ± 6.90	26.29 ± 6.67	25.52 ± 6.05	−5.22 (16.6%)	−5.99 (19.0%)	0.48	0.41
	4.2 ± 2.7	4.8 ± 0.3	4.5 ± 0.7	+0.6 (14.2%)	+0.3 (7.1%)	0.86	0.92
LC7	30.22 ± 2.53	29.00 ± 3.85	28.82 ± 2.53	−1.22 (4.0%)	−1.40 (4.6%)	0.66	0.52
	8.9 ± 4.7	15.6 ± 3.7	11.6 ± 5.6	+6.6 (73.3%)	+2.6 (28.9%)	0.09	0.54
LC8	31.43 ± 5.94	28.10 ± 6.97	27.37 ± 8.27	−3.33 (10.6%)	−4.06 (12.9%)	0.55	0.52
	6.8 ± 4.7	8.5 ± 5.7	8.3 ± 4.8	+1.7 (25.0%)	+1.5 (22.1%)	0.76	0.77

Data are given in g/hm^2^ for TEWL and in Corneometer^®^ CM 825 arbitrary units (a.u.) for skin capacitance, i.e., skin hydration (*n* = 4; data are shown as mean ± standard deviation (SD)). ^#^ Average change from baseline in absolute (g/hm^2^ for TEWL and a.u. for skin hydration) and relative (%) values. *p*-value *—change from baseline after 30 min. *p*-value **—change from baseline after 90 min.

**Table 4:** Interlayer spacing *d* (calculated from the first peak top of the small-angle X-ray scattering (SAXS) curves) of the tested LC1–LC8 at given temperatures.

Sample	*d* (nm)		
	25 °C	32 °C	37 °C
LC1-AP	8.76	8.64	8.58
LC2-AP	7.74	7.75	7.76
LC3-AP	8.27	8.34	8.28
LC4-AP	8.87	8.96	9.02
LC5-AP	9.87	10.10	10.22
LC6-AP	10.99	11.13	11.63
LC7-AP	11.24	12.00	12.50
LC8-AP	/	/	/

**Table 5:** Viscosities (mean ± S.D. (*n* = 2)) at 25, 32, and 37 °C and loss tangent (tan δ) at frequency 100 Hz for the tested LC1-AP to LC8-AP samples with corresponding water/surfactant (W/S) ratio.

Sample	W/S Ratio	η (Pas) 25 °C	η (Pas) 32 °C	η (Pas) 37 °C	tan δ (100 Hz)
LC1-AP	0.36	47.3	44.8	43.4	0.333
LC2-AP	0.48	18.9	9.2	8.3	0.497
LC3-AP	0.61	34.3	30.6	22.2	0.166
LC4-AP	0.78	26.6	17.5	16.0	0.147
LC5-AP	0.95	81.2	37.4	28.7	0.090
LC6-AP	1.18	73.3	42.2	32.3	0.089
LC7-AP	1.43	155.0	108.9	81.1	0.070
LC8-AP	1.77	70.4	59.1	52.4	0.044

**Table 6:** The composition of tested AP-loaded LCs lies on the same dilution line (m/m%).

Sample ^#^	(Tween 80/Lecithin) *	IPM	Bidistilled Water	AP
LC1-AP	55.44	23.76	19.80	1
LC2-AP	51.48	22.77	24.75	1
LC3-AP	48.51	20.79	29.70	1
LC4-AP	44.55	19.80	34.65	1
LC5-AP	41.58	17.82	39.60	1
LC6-AP	37.62	16.83	44.55	1
LC7-AP	34.65	14.85	49.50	1
LC8-AP	30.69	13.86	54.45	1

^#^ For LC systems without AP incorporated, the samples were prepared to keep the same mass ratio between the components as presented (Tween 80/lecithin/IPM/water). * Mass ratio of Tween 80 to lecithin is 1/1.

## Data Availability

The data presented in this study are available in article and Supplementary Materials.

## Supplementary Materials

The following supporting information can be downloaded at: https://www.mdpi.com/article/10.3390/molecules29133173/s1.
