# Supplementary material for: The Impact of Phospholipid-Based Liquid Crystals’ Microstructure on Stability and Release Profile of Ascorbyl Palmitate and Skin Performance
Source: Molecules. 2024 Jul 3;29(13):3173. doi: 10.3390/molecules29133173 (PMC11243444; doi:10.3390/molecules29133173)
Supplement: Supplementary file 1 [file molecules-29-03173-s001.zip › molecules-3061701-supplementary.pdf]

**SUPPLEMENTARY DATA for the study**

# The impact of phospholipid-based liquid crystals' microstructure on stability and release profile of ascorbyl palmitate and skin performance

**Alenka Zvonar Pobirk[1], Robert Roškar[1], Marija Bešter-Rogač[2], Mirjana Gašperlin[1], Mirjam Gosenca Matjaž[1]**

[1] University of Ljubljana, Faculty of Pharmacy, Aškerčeva cesta 7, 1000 Ljubljana, Slovenia

[2] University of Ljubljana, Faculty of Chemistry and Chemical Technology, Večna pot 113, 1000 Ljubljana, Slovenia

**Table S1:** Area under the curve (AUC) obtained by 2 subsequent injections of samples loaded with ascorbyl palmitate in high-performance liquid chromatography (HPLC) analysis.

| Sample | AUC (1st injection) | AUC (2nd injection) |
|---|---|---|
| **LC1** | 598,2 | 586,7 |
| **LC8** | **475,0** | **467,4** |
| **SMEDDS** | 616,6 | 605,5 |
| **Isopropyl myristate** | 773,3 | 680,5 |
| **Microemulsion** | 617,4 | 567,4 |

*LC (liquid crystal); SMEDDS (self-microemulsifying drug delivery systems)*

**Table S2:** Average values of Area under the curve (AUC) for various ascorbyl palmitate (AP) solutions immediately after preparation and after 24 h with different antioxidants and solvents tested to improve the stability of AP.

| Solution | Time = 0 | Time = 24h | Stability (%) |
|---|---|---|---|
| **AP Standard solution in methanol** | 642,0 | 423,3 | 65,9 |
| **AP Standard solution + EDTA in methanol** | 724,7 | 662,4 | 91,4 |
| **AP Standard solution + ascorbic acid in methanol** | 671,1 | 645,6 | 96,2 |
| **AP Standard solution + BHT in methanol** | 704,1 | 407,8 | 57,9 |
| **AP Standard solution in acetonitrile** | 606,5 | 164,0 | 27,0 |

*BHT (butylated hydroxytoluene); EDTA (ethylenediaminetetraacetic acid)*

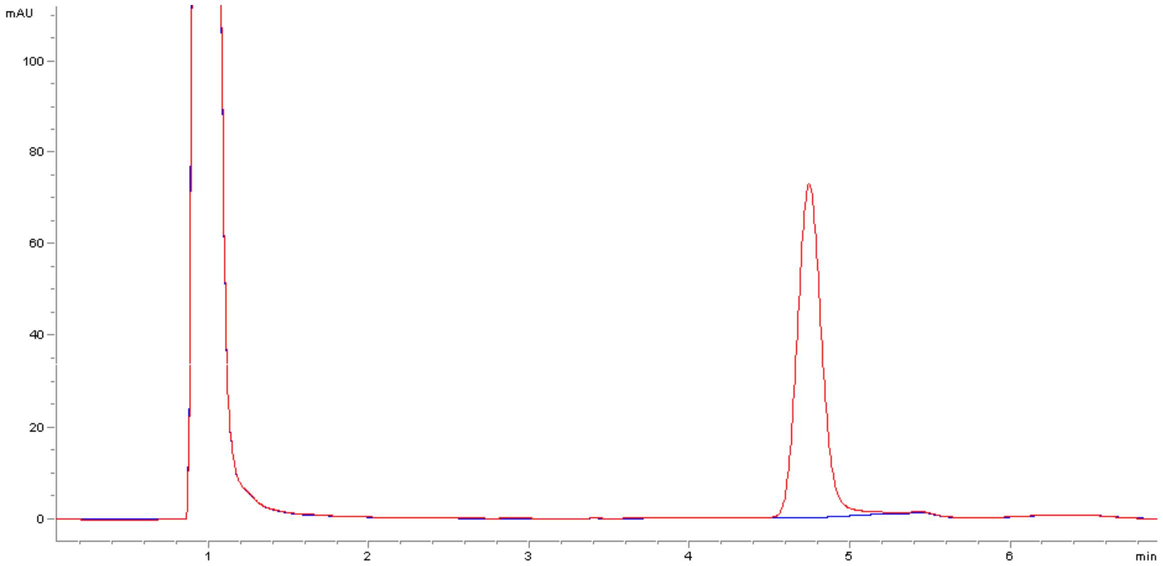


**Figure S1.** Superimposed chromatograms for plain sample LC (blue curve) and AP-loaded LC1 (red curve) dissolved in methanol with ascorbic acid added in 200 mg/L concentration. The peak with retention time at approx. 4,8 min corresponds to AP, whereas the peak with retention time of approx. 1 min corresponds to ascorbic acid added as stabiliser.

**Table S3.** Validation data for HPLC method.

| SAMPLES | Sample number | AUC | Average AUC | SD | RSD (%) |
|---|---|---|---|---|---|
| **LC1 (with AP)** | 1 | 602,4 | 601,8 | 2,4 | 0,39 |
| | 2 | 604,3 | | | |
| | 3 | 603,0 | | | |
| | 4 | 598,1 | | | |
| | 5 | 601,0 | | | |
| **LC1* (with AP)** | 1 | 763,7 | 762,1 | 2,1 | 0,28 |
| | 2 | 759,7 | | | |
| | 3 | 762,9 | | | |
| **LC1 (with AP + AP standard solution)** | 1 | 1516,4 | 1531,0 | 12,9 | 0,84 |
| | 2 | 1535,8 | | | |
| | 3 | 1540,9 | | | |
| **AP standard solution** | 1 | 918,8 | 926,4 | 9,6 | 1,03 |
| | 2 | 923,3 | | | |
| | 3 | 937,2 | | | |
| **Accuracy (%)** | | | **100,3** | | |
| **Stability (%) 1d** | | | **95,0-103,4** | | |

** different sample as used in repeatability testing*

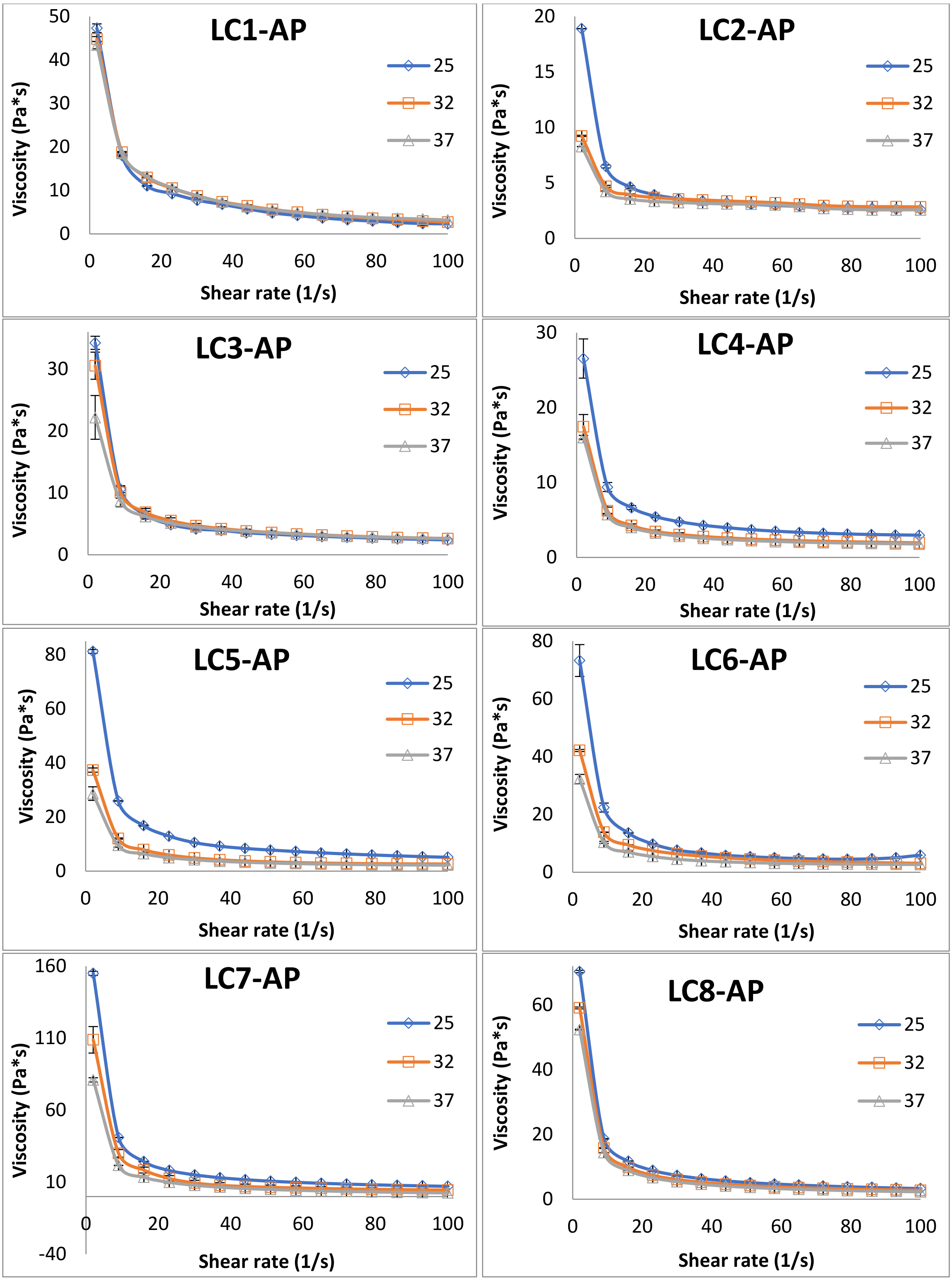


**Figure S2:** Viscosity flow curves for LC-AP1 to LC-AP8 measured at 25, 32 and 37 °C.
